# Immunoglobulin G in aging and cancer

**DOI:** 10.3389/fimmu.2026.1854902

**Published:** 2026-07-03

**Authors:** Matthew Bu, Zhongguang Li, Elyse Jones, Umme Lubaba, Xuefeng Liu, Haichang Li

**Affiliations:** 1 Department of Veterinary Biosciences, College of Veterinary Medicine, The Ohio State University, Columbus, OH, United States; 2 The Ohio State University Comprehensive Cancer Center, Columbus, OH, United States; 3 Departments of Pathology, Urology and Radiation Oncology, Wexner Medical Center, The Ohio State University, Columbus, OH, United States; 4 Department of Surgery, The Wexner Medical Center, The Ohio State University, Columbus, OH, United States

**Keywords:** aging, cancer, glycosylation, immunoglobulin g (IgG), inflammation

## Abstract

Immunoglobulins are key effector molecules in humoral immunity and play fundamental roles in human health and disease through their complex structure-function relationships. Dysregulation of immunoglobulin production or function underlies numerous conditions, including inflammatory disorders, autoimmune diseases, and metabolic diseases. In the context of cancer, immunoglobulin G (IgG) is frequently enriched within tumor tissue and displays aberrant glycosylation, where it correlates with cancer progression, and its functional impact within the tumor microenvironment is drawing growing interest. Furthermore, recent studies have collectively identified as a novel aging-associated factor involved in multi-organ aging processes. Understanding the role and the mechanisms of immunoglobulins in both physiological and pathological conditions remain a major priority for researchers. Elucidating the underlying pathophysiology and mechanisms of IgG may therefore provide valuable insights for the development of new therapeutic strategies. In this review, we summarize recent progress in the study of IgG in aging and cancer and discuss its potential roles in the future landscape of immunology and biomedical research.

## 1 Introduction

Immunoglobulins, alternatively known as antibodies, are central to adaptive immunity, and mediate host defense through pathogen neutralization, complement activation, and Fc receptor based cellular responses (1, 2). Among them, Immunoglobulin G (IgG) is the predominant isotype in circulation and plays key roles in modulating immune cell activity as an effector molecule (2). IgG assumes much of its effector function from its Fc glycosylation, which may then influence its binding interactions with Fcγ receptors (3). Through these mechanisms, IgG participates in the regulation of immune homeostasis under both physiological and pathological conditions (4).

Aging is accompanied by widespread remodeling of the immune system, including the expansion of age-associated B cells (ABCs) and their atypical antibody repertoire (5). The IgG isolated from older individuals often shows characteristic changes in Fc glycosylation, most notably a reduction in galactosylation and sialylation (6). These resulting changes are then seen to favor activating Fcγ receptors interactions, thus associating with enhanced pro-inflammatory signaling (7). Furthermore, the age-associated IgG is seen to affect macrophage dynamics permitting the persistence of chronic low-grade inflammation, a defining feature of aging known as inflammageing (8, 9). Altogether, these findings support a potential role for IgG in contributing to the progression of an inflammatory environment during aging.

Aging is also a major risk factor towards developing cancers, in which IgG may display impaired functions, given the immunomodulatory nature of cancers (10). In expansion, immunoglobulin, particularly IgG, is frequently found at elevated levels within tumor tissue, where it has been associated with cancer progression; this intratumoral pool, here termed tumor-associated IgG (T-IgG), is most likely contributed by tumor-infiltrating B cells and plasma cells (11). T-IgG differs from traditional B cell derived IgG in its glycosylation and consequently, its binding partners (12, 13). These alterations then allow T-IgG to act locally within the tumor microenvironment (TME) to support cell survival, metabolic adaptations, and tumor metastasis (14–16).

Despite progress in both aging and tumor immunology, the impact of IgG modifications in both age-related and T-IgG, and their relation remains unclear. Here, we propose that IgG remodeling during aging establishes a pro-inflammatory baseline (7), that can be further exploited in cancer pathology and is exacerbated by elevated intratumoral IgG (17). Similarities between age-associated IgG and T-IgG include altered glycosylation, receptor engagement, and downstream signaling, suggesting a functional overlap that warrants further study (18, 19). In this perspective IgG remodeling is framed as a potential basis that links the immunological landscape of aging and tumorigenesis.

By synthesizing these fields, we aim to map how age-associated and T-IgG converge to potentially promote a tumor permissive environment, while assessing their clinical utility as diagnostic biomarkers and therapeutic targets (20–22). To establish a unifying conceptual framework for these context-dependent adaptations, **Table 1** delineates the key structures, receptor engagement patterns, and downstream outcomes that distinguish physiological, age-associated, and T-IgG states.

**Table 1:** Structural and functional differences in IgG.

Dimension	Physiological IgG	Age-Associated IgG	T-IgG
Origin	Plasma cells	Age-associated B cells (ABC)	Likely tumor-infiltrating B/plasma cells
Glycan Signature	Balanced Fc galactosylation, fucosylation, and sialylation at Asn 297	Agalactosylation and hyposialylation at Asn 297	Atypical glycan at Asn 162 (CA215 epitope) terminal sialylation
Receptor Axis	Balanced FcγR engagement	Activating bias toward activating FcγR and enhanced FcRn-mediated recycling	Non-canonical receptor engagement with integrin, α6β4, c-Met, and inhibitory Siglecs
Functional Output	Immune surveillance; balanced ADCC and CDC; efficient pathogen clearance	Chronic inflammation; macrophage dysfunction; tissue fibrosis	Tumor-promoting programs: stemness, EMT, metabolic reprogramming, and immune evasion
Microenvironmental Impact	Tissue homeostasis	Pro-inflammatory niche and SASP amplification	Immunosuppressive tumor microenvironment
Mechanistic Driver	Stable Fc glycosylation and regulated FcγR signaling	Glycan remodeling (G0 shift) and chronic immune activation	Aberrant glycosylation and receptor rewiring
Translational Implication	Benchmark for humoral immunity; foundation of therapeutic monoclonal antibodies	Biomarkers of biological aging; targets for glycoengineering interventions	Emerging targets in precision oncology: diagnostics (e.g., RP215) and immunotherapy strategies (e.g., CAR-T)

## 2 Age-associated B cell and IgG driven immune dysregulation

The advancement of age is often viewed synonymously with the progressive deterioration of molecular interactions that result in the impairment of overall tissue function (23). B cells are no exception to this pattern and progressively lose functionality over time, as evidenced by the reduced IgG diversity, impaired class switching, and diminished antibody affinity (5). Canonically, a humoral immune response is led by the production of pentameric IgM in response to initial pathogenesis (24). However, due to its relatively inert binding specificity, prolonged antigen exposure prompts specific B cell expansion and class-switching to yield the monomeric IgG, which strongly mediates opsonization, antigen sequestration, and downstream effector functions (3). Interestingly, the increased incidence of age-associated IgG is corroborated by a similar trend in the accumulation of IgM in older populations, which has been recently proposed as an independent biomarker of aging (25). The observed increase of both IgM and IgG in aged tissue underscore a systemic shift in the B cell repertoire, insinuating a resurgence of unintentional B cell activation, leading to a pro-inflammatory milieu preceding oncogenesis. The continuous decline of the B cell population may then contribute to the host mounting deficient immune responses against pathogens, as well as contributing to autoimmunity (26). Because of the modified state of these B cells, the resulting antibody production is similarly divergent from the juvenile phenotypes. IgG, in particular, is noticeably altered with respect to its N-glycan patterning (27). As the glycan profile of IgG changes throughout age, the Fc binding function can be substantially altered, stimulating pro-inflammatory Fcγ receptors such as FcγRIIIa and FcγRIIa (18, 28). Thus, it is important to investigate the influence of age-associated IgG, particularly regarding abnormal engagement with Fcγ receptors and the consequent downstream inflammatory signaling. Additionally, the efficacy of immunoglobulin-mediated immunity is reduced in aging, with diminished antibody class representation, compounded by the loss of Fab affinity (29). The tendency of immunoglobulins towards pathology is especially heightened in the elderly, which is thought to be partially due to the enriched population of differentiated, age-associated, B cells (ABCs) (30, 31).

### 2.1 Age-associated B cell expansion: a source of pathological IgG

First to understand the precise role of age-associated IgG in aging, it is necessary to examine the primary cellular machinery responsible for its production in B cells. The ABC repertoire undergo a gradual shift in aging, marked by their progressive accumulation, and tendency to exhibit distinct immunosenescent profiles in addition to increased expression of FcγRIIb and IL-1R, the latter of which being cited as an NLRP3 inflammasome mediated promotor of B cell expansion (31, 32). Furthermore, the intracellular machinery in these aged B cells is fundamentally altered, potentially through tristetraprolin mediated destabilization of E47 mRNA transcription, resulting in reduced activation induced cytidine deaminase activity (33). This observation, in turn, may partially explain the impaired class-switching ability in the elderly, and the associated enrichment of pathological IgG in aging.

Ultimately, it is hypothesized that the ABC population serves as a potential cellular source of IgG seen to negatively impact the aging process (34). While it is well-established that ABCs can produce highly inflammatory and autoreactive IgG, direct evidence linking the intrinsic enzymatic machinery in ABCs to the specific glycosylation profile of age-associated IgG remains an active area of investigation (18, 35). Regardless, by suppressing healthy B cell proliferation, these ABCs dictate the consequent humoral landscape and indirectly support the accumulation of age-associated IgG.

A potential mechanistic reasoning for this deliberate expansion of the aged B cell cohort is substantiated by elevated inflammatory tone stemming from environmental SASP triggers (36). Early studies demonstrate that TNF-α induces apoptosis selectively in physiological, bone-derived pro-B cells, allowing ABCs to dominate the B cell population (37). This dynamic likely reflects a feedback loop, wherein baseline inflammatory cytokines such drive the expansion of ABCs, resulting in the aggregation of pathogenic IgG substrates, which in turn intensifies pre-existing dysregulated conditions. Although the direct molecular sequence governing this cycle remains theoretical, this proposed reciprocal relationship between ABC expansion and inflammageing represents a potential driving factor in age-related IgG synthesis.

### 2.2 Age-associated IgG drives tissue senescence and fibrosis

Once established, these ABC clusters may concurrently express age-associated IgG, which acts to further aggravate pre-existing inflammation. IgG was abundantly detected within pro-senescence hotspots via analysis of a novel spatial transcriptome, suggesting that IgG depots may correlate to aging as a potential evolutionary correlate (38). Furthermore, the IgG-containing senescent hotspots were closely tied to several aging hallmarks expressed by neighboring cells such as SA-β-Gal, P21, and γH2AX (38). When taken into consideration with an increased expression of NF-κB, STAT1, and iNOS in age-associated IgG-treated macrophages, it is likely that IgG may actively contribute to cellular senescence and the preservation of inflammageing (38).

This dynamic reinforces the potential feed-forward loop wherein baseline inflammatory signaling drives the expansion of the ABC population (37). Consequently, these ABCs aberrantly secrete a specific age-associated IgG pool that is sufficient to stimulate local tissue senescence and potent inflammatory mediators including CCL2, IL-6, and TNF-α, thereby perpetuating inflammageing (9). These findings are corroborated by previous literature demonstrating IgG immune complex-mediated engagement with FcγRIIa on peripheral blood mononuclear cells to produce TNF-α in rheumatoid arthritis synovial fluid (28).

**Figure 1:**
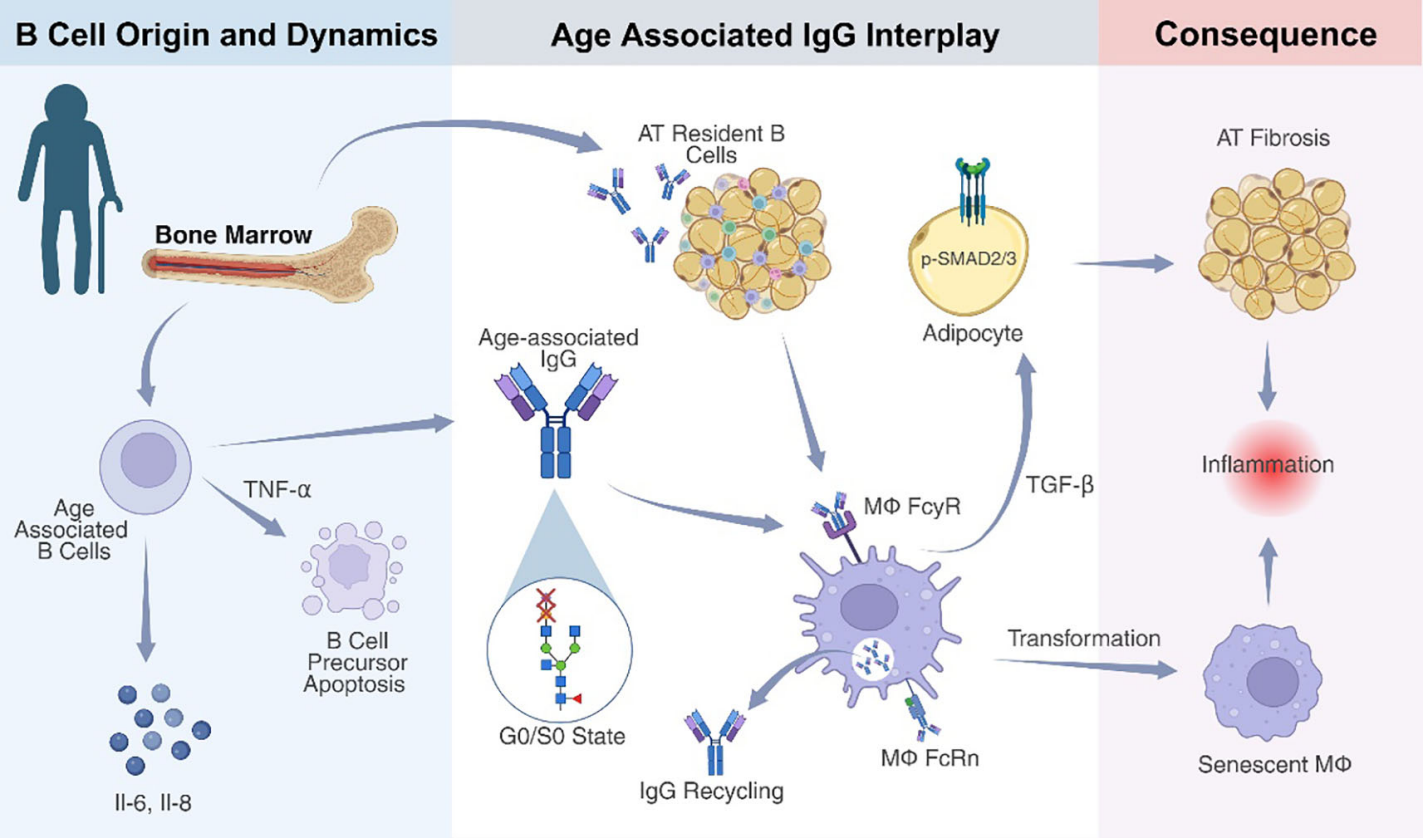
Age-associated IgG derives macrophage senescence and FcγR-mediated adipose tissue Fibrosis. Depicted is the accumulation of age-associated B cells which release pro-inflammatory cytokines and are maintained through TNF-α-mediated suppression of B cell precursors (5, 37). Additionally, the age-associated IgG products may act upon neighboring macrophages to activate FcγR-mediated TGF-β signaling to induce fibrosis in adipose tissue and promote macrophage senescence (9, 38). The age-associated IgG is also recycled by macrophage FcRn receptors to prolong their pathogenic effects (39).

Moreover, recent studies have implicated age-associated IgG as a plausible catalyst in adipose tissue fibrosis and metabolic dysfunction (**Figure 1**) (9). Macrophages, as key mediators of chronic inflammation and age-related disease development, are again highlighted in this process (38, 40). IgG isolated at various timepoints across the murine lifespan was administered to bone-derived macrophage, resulting in an IgG-dependent increase in TGF-β/SMAD mediated AT fibrosis (9). Furthermore, the accumulation of B cells in visceral adipose tissue and class-switched IgG may affect insulin resistance through the amplification of macrophage FcγR binding and subsequent cytokine release (41). These effects are also prolonged by the recycling of IgG via FcRn receptors on macrophages, allowing the IgG to continually stimulate AT fibrosis, metabolic dysfunction, and inflammageing (39, 42). In summary, a specialized, pro-inflammatory, subset of aged adipose tissue B cells is a potential mediator of chronic low-grade inflammation and homeostatic imbalances, producing age-associated IgG capable of influencing the tissue microenvironment.

Beyond the immediate contributions of age-associated IgG in the form of inflammageing and metabolic dysregulation, there is also evidence linking age-associated antibodies to the progression of age-related autoimmune diseases, such as multiple sclerosis, rheumatoid arthritis, and Sjögren’s syndrome (43, 44). These claims are further supported by the gradual increase of ABCs seen producing autoreactive IgG through aging, accompanied by greater markers of inflammation, signaling the possible occurrence of pathogenic autoimmunity (45). A main point of convergence in many of the various autoimmune diseases containing high levels of ABCs is the presence of pathogenic class-switched IgG (46). T-bet, a transcription factor often expressed by ABC subsets, is consistently tied to the production of highly inflammatory and pathogenic IgG2a in mouse strains bred with autoimmune diseases (47). Similarly, T-bet is linked to Ig class-switching towards IgG1 in humans, as shown in T-bet positive B cells upon viral infection (48).

These findings suggest that T-bet expressing ABC subsets could mediate pathogenic IgG class switching in autoimmune pathology. However, with the incomplete state of the research in human models, as well as the lack of research directly associating age-associated IgG with self-reactivity, it is vital to approach these findings cautiously (18). Future directions should focus on defining the relationship between ABCs and age-associated IgG by studying their effects both independently and synergistically to determine their relative roles in pathology and aging as well as revealing any potential interdependencies. Importantly, much of the age-associated IgG activity is due in part to its structural modifications, such as N-glycosylation, which regulate Fc receptor engagements (18).

## 3 Regulation of IgG glycosylation in aging

The immunomodulatory capacity of IgG is dictated by the complex glycosylation profiles located on both the Fc and Fab regions (27, 49). While this review primarily examines the highly conserved N-linked glycan attached to the Asn 297 residue of the Fc region, it is of critical importance to acknowledge the physiological role of Fab glycosylation. Unlike the canonical Fc glycosylation, which primarily modulates effector binding and function, the Fab glycans typically influence antibody recognition capacity by modifying binding affinity, stability, and specificity (49). Furthermore, the deviation in the Fc glycome is not known to be particularly mirrored in the Fab region, highlighting a potential difference in regulation of IgG glycosylation by region (50). In the context of malignancy, Fab glycosylation is not well documented. However, emerging evidence suggests that Fab glycosylation may facilitate tumor cell survival, by impairing antigen-antibody interactions and macrophage polarization (51, 52). Understanding the systemic balance between Fab and Fc glycosylation is essential, as alteration to either domain are potential sources for dysregulation of IgG function.

Pertaining to the structure of the Asn 297 glycan chain, the addition or removal of sugar moieties, such as fucose, galactose, and sialic acid may then alter the Fc region conformation, thereby altering its FcγR receptor engagements (35). During aging, the glycosylation patterns deviate, contributing to a shift towards pro-inflammatory glycoforms (18). This heterogeneous, age-associated glycome remodeling is comprised of a complex interplay of intrinsic and extrinsic factors, encompassing highly heritable genetic traits linked to B cell transcription factors (e.g., RUNX1, IKZF1), as well as the activity of extracellular glycosyltransferases (53, 54).

### 3.1 IgG galactosylation in aging

Among the various IgG N-glycans, galactosylation has been studied extensively, with agalactosylation (G0) present in a myriad of diseased states (55, 56). Furthermore, G0 glycoforms have been associated with a pro-inflammatory phenotype, which is problematic given the decline in IgG galactosylation observed with aging (57). In contrast, younger individuals normally display adequate IgG Fc galactosylation of either a single branch (G1) or of both terminal branches (G2), with loss of galactosylation being seen to correlate with increased risk for age-related pathologies, such as ischemic stroke (20, 58). Galactosylation state also influences complement activation in innate immunity, with specific glycan changes regulating complement-dependent cytotoxicity (59). The current theory is that the complement system is regulated through IgG interaction with Dectin-1 and FcγRIIb, downplaying C5a-mediated inflammatory pathways (60, 61). However, the mechanism of action associated with agalactosylated IgG has not been completely defined, underscoring the need for additional evidence linking agalactosylated IgG to age-related health decline. Similarly, terminal sialylation has been reported to decrease with age, although findings remain heterogenous (20, 62).

### 3.2 IgG sialylation in aging

Despite this, sialylation is increasingly discussed in gerontology, as loss of sialylation (S0) has been observed in association with pro-inflammatory states (63). Notably, Tanigaki et al. demonstrated that hyposialylated IgG activates endothelial FcγRIIb in promotion of obesity-related pathogenesis, highlighting the potential pathological effects of S0 in the aged population (64). In comparison, increased sialylation of IgG Fc region is reported to reduce antibody-dependent cellular cytotoxicity (ADCC) via a reduction of FcγRIIIa binding affinity on natural killer (NK) cells, which may be lost in aging (65). The manner in which IgG Fc sialylation relays its effects is not fully known, but some predict that it depends upon an α-2,6-sialylation linkage, potentially enabling participation in IL-33-dependent FcγRIIb upregulation (61, 66). Moreover, Chakraborty et al. discovered an important interaction between Fc-sialylated anti-influenza IgG and NF-κB repression via the REST induction, further revealing the potent anti-inflammatory properties of Fc-sialylated IgG (67). Together, these findings suggest that changes in IgG Fc glycosylation may selectively alter inflammatory signaling during aging, necessitating investigation into additional glycan residues and their regulatory effects.

### 3.3 Core fucosylation and bisecting GlcNAc alterations in aging

Another important residue present on the Asn Fc glycan is the core fucose, which is known to substantially suppress ADCC when present (27). Additionally, enriched afucosylation in IgG was tied to the release of pro-inflammatory cytokines IL-6 and TNF-α, likely through FcγRIIIa ITAM signaling (68). Interestingly, the bisecting GlcNAc is often inversely correlated to core fucose and is proposed to function in a contradictory manner. However, it remains unclear whether the effects are causative or merely correlative, given the limited literature accounting for the frequent co-occurrence of core fucose and bisecting GlcNAc (18). Despite this, the combination of non-galactosylation and bisecting GlcNAc has been associated with aging, with the reduction of this glycoform being positively associated with longevity, potentially through GlcNAc transferase III activity (69). Overall, the ABC-derived IgG cohort not only reflects immunosenescence but also represents a state of heightened vulnerability, particularly in pathological contexts such as autoimmunity and metabolic disease (9, 55). Furthermore, given that age is the strongest risk factor for malignancy, it is important to understand how age-associated deviations in IgG structure and function may influence tumor biology (10). Future studies should therefore investigate whether age-associated IgG actively contributes to carcinogenesis or tumor progression.

## 4 Tumor-associated IgG and its role in cancer progression

IgG is frequently elevated within tumor tissue across many epithelial malignancies (70, 71). This increased intratumoral IgG consistently correlates with advanced stage, greater metastatic propensity, and poorer overall survival (72, 73), prompting interest in its functional contribution to tumor biology.

Rather than mediating systemic pathogen clearance like conventional antibody, T-IgG appears to act locally within the TME, where it has been linked to tumor cell survival, metabolic adaptation, and proliferative signaling (74). This positions it as a potential active contributor to cancer progression and a candidate prognostic indicator, with the underlying mechanisms discussed in the following sections.

Importantly, the IgG detected in tumors is most likely produced by tumor-infiltrating B cells and plasma cells, which are abundant in the TME and are the established source of secreted antibody (11). Earlier reports attributing this IgG to tumor cells themselves relied on methods that did not rigorously exclude B-lineage contamination, and this interpretation remains controversial (75). Critically, these functional and prognostic associations are unaffected by this question of origin and remain relevant even if the IgG derives entirely from infiltrating B cells.

### 4.1 Structural divergence and subcellular localization

Regardless of its cellular source, the immunoglobulin enriched in tumors feature unique structural and genetic properties such as distinct recombination patterns and atypical glycosylation (75). Immunoglobulin sequences recovered from tumor preparations reportedly show restricted, oligoclonal V(D)J rearrangements rather than a diverse B-cell repertoire (74, 76). Since V(D)J recombination is a defining, RAG1/2-dependent feature of B-lineage cells, this is most simply explained by a clonally limited infiltrate of B or plasma cells, not by recombination in epithelial tumor cells (11). A defining feature of T-IgG is the atypical Asn162 N-glycosylation, which is thought to facilitate non-canonical receptor interaction (12). T-IgG is prevalent within the TME, both at the tumor cell surface and as a secreted component, under the influence of the local environment (17, 77, 78).

### 4.2 Hijacking growth and stemness pathways

**Figure 2:**
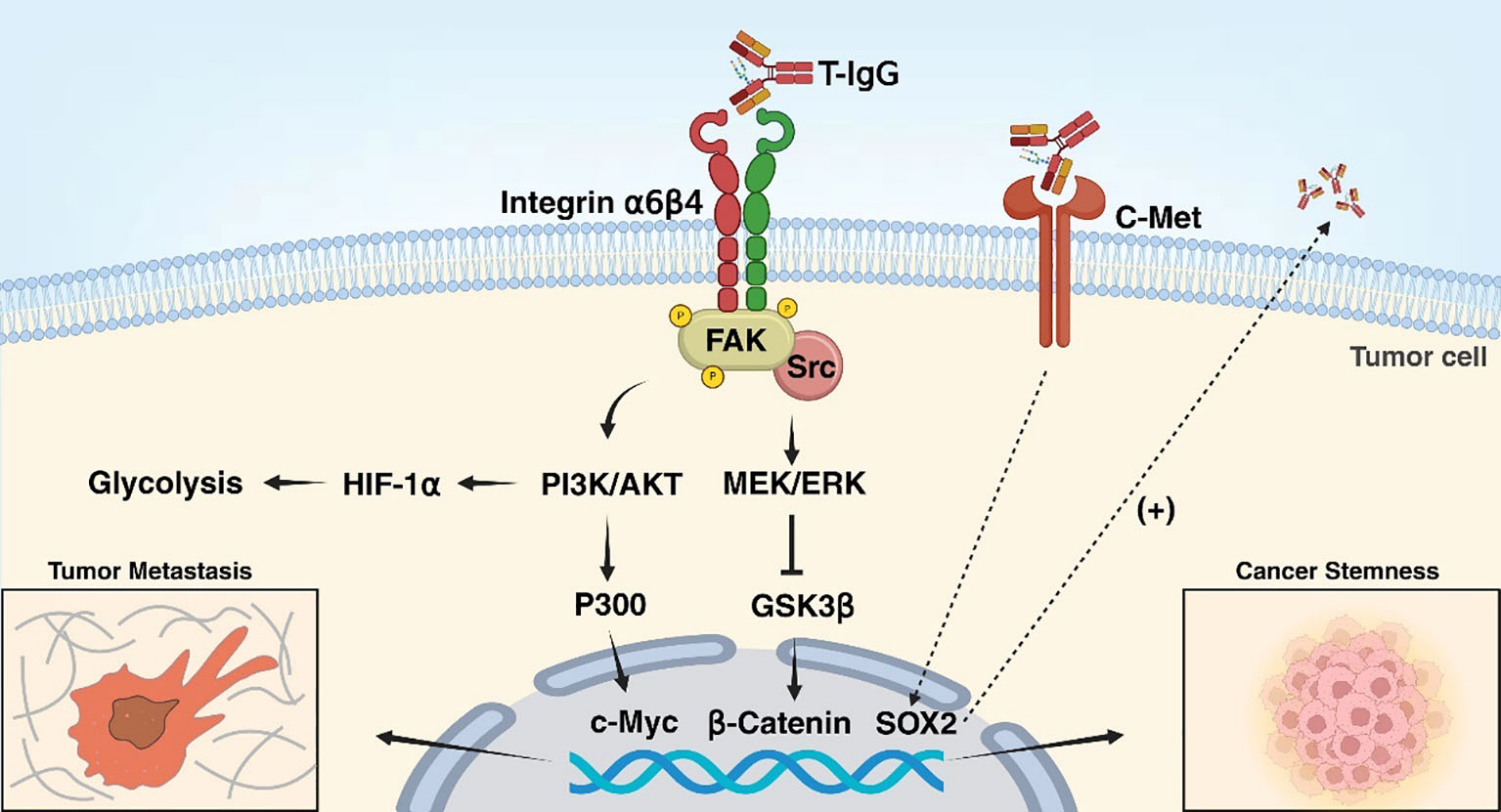
Tumor-associated IgG promotes oncogenic signaling. Proposed signaling pathways involving tumor- associated IgG (T-IgG) binding to integrin α6β4, followed by FAK/Src-mediated recruitment of downstream PI3K/AKT and MEK/ERK signaling cascades (12, 80, 82). These pathways converge on transcription factors c-Myc and β-catenin which may support cancer progression (83, 84). The diagram also illustrates the reported effects of T-IgG on cancer cell metabolism via HIF-1α, as well as a possible positive feedback mechanism between T-IgG and c-Met (13, 85).

One of the hallmarks of cancer is its ability to bypass cellular checkpoints present in healthy cells (79). Determining the fundamental basis behind the hijacking of cellular processes is central to understanding carcinogenesis from broader perspectives and may even deepen our understanding of senescence in aging. T-IgG is again relevant in this context, with reports of heavy chain encoding IGHG1 potentially contributing to colony formation, tumor growth in mice, and stemness markers in culture (13, 15, 80). IGHG1 expression in prostate cancer has been linked to enhanced proliferation, potentially through SOX2-mediated upregulation of T-IgG signaling (81). Consistent with these findings, a novel c-Met/SOX2 feedback loop has been proposed, with T-IgG engaging receptor c-Met in a novel binding interaction (**Figure 2**) (13). This interaction was associated with elevated expression of transcription factors OCT4 and SOX2, downstream of PI3K/AKT and MEK/ERK signaling, suggesting a potential mechanistic basis for the acquisition of stem-like rejuvenation (13).

### 4.3 T-IgG mediated glycolytic shift and redox homeostasis

Beyond T-IgG’s influence on proliferative signaling, emerging data suggest that T-IgG may play a role in reprogramming metabolism within tumor cells. Wang et al., provided initial insight by demonstrating T-IgG’s complexation with RACK1, RAN, and PRDX1, shifting redox metabolism towards a low-grade ROS status permissive to oncogenesis (86). However, as an early mechanistic study, the *in vitro* systems leave considerable uncertainty regarding T-IgG’s role and whether T-IgG acts as an accessory factor within the pro-oxidative cascade or as an upstream mediator. More recently, attention has turned to a direct connection between T-IgG and metabolic processes in triple-negative breast cancer through a proposed role in promoting glycolytic metabolism (85). Glycolytic enzymes HK2 and LDHA, as well as ATP production, were shown to be dysregulated in T-IgG knockdown models. In addition, MCT-1 and MCT-4 expression were also significantly altered upon T-IgG modulation, indicating its possible involvement in the transport of lactate in support of tumor growth (85).

### 4.4 Adhesion networks and metastatic dissemination

Emerging data also suggest T-IgG may serve a role in tumor dissemination, epithelial-mesenchymal transition (EMT) and vascularization via activation of the TGF-β/SMAD signaling cascade (15, 82, 87). A key mechanistic insight into T-IgG-mediated tumorigenesis was first proposed by Tang et al., demonstrating that non-canonically glycosylated IgG binds integrin receptor α6β4 and its co-receptor CD44 in lung cancer (12). Interestingly, the Asn 162 glycosylation does not appear to mediate initial binding yet is crucial for autophosphorylation of FAK and downstream cortactin/paxillin dynamics, suggesting that T-IgG harbors additional deviations beyond its N-glycosylation (12). Moreover, the Wnt signaling pathway may be linked to T-IgG activity, whereby PI3K/AKT-mediated phosphorylation of GSK3β stabilizes β-catenin following initial integrin/FAK/Src binding (84). Importantly, T-IgG has been shown to bind FcγRIIa receptors on circulating platelets, potentially conferring motility and immune protection to disseminating tumor cells (88).

### 4.5 Current mechanistic limitations and perspectives

Altogether, the current research points to T-IgG as a key intermediary molecule in cancer progression. Through the reprogramming of metabolic machinery, the T-IgG has been proposed to alter cellular plasticity and migratory capacity through FAK and c-Met binding (12, 13). However, due to the novelty of the field, current literature relies heavily on proof-of-concept *in vitro* models, with limited *in vivo* validation and little mechanistic cross-verification (75). As a result, the hierarchical relationships among these mechanisms in tumor growth and metastasis remain unclear, requiring further studies to determine whether T-IgG is a primary driver of migration and stemness acquisition or a context-dependent effector within established tumorigenic networks. Future studies should prioritize in-depth characterization of receptor-ligand interactions, and integrative genomic-proteomic analysis across a heterogeneous panel of cancer cell lines to elucidate the signaling networks through which T-IgG contributes to tumor growth and metastasis. Importantly, future studies employing rigorous lineage-tracing approaches, single-cell sequencing, and spatial transcriptomic analyses will be required to definitively establish the cellular origin of T-IgG and clarify its contribution to tumor progression.

### 4.6 Immune evasion and therapeutic resiliency

Throughout the past decade, there has been growing evidence suggesting a role for T-IgG in tumor progression both as a competitive ligand and through its effector function (14, 89). Early studies reported reduction in apoptotic caspase markers and increased proliferation with T-IgG overexpression (90). These findings suggest that T-IgG may suppress immune signaling in the host, an effect that could be amplified at the tissue level, thereby fostering a tumor microenvironment conducive to immune evasion and tumor growth. Natural killer (NK) cells, as the first-line defense against cancer, are frequently impaired in malignancy (91). Among the many mechanisms that drive NK downregulation, T-IgG has been implicated in reducing ADCC when tumor cells are co-cultured with NK cells (92). While the direct signaling pathway remained unconfirmed, it is possible that the circulating T-IgG competitively occupies NK Fc receptors. The theory is supported by the co-localization of complement proteins C1q, C3, C4, and T-IgG in papillary thyroid carcinoma, suggesting the formation of immune complexes that may facilitate immune evasion (93). The adaptive immune response may also be irregularly modified by T-IgG, with growing evidence of its preferential interactions with inhibitory Siglec-7 and Siglec-10 (14, 94). However, the validity of this anergic signaling response remains unclear, prompting further research to focus on clarifying the mechanism of T-IgG in Siglec-dependent immune modulation. By inhibiting NK cell activity, co-localizing with complement proteins, and selectively binding inhibitory Siglecs, T-IgG may exert suppressive pressure on the host immunity, allowing the TME to persist and promote tumor immune evasion (14, 92, 93).

Beyond tumor progression, T-IgG has also been associated with therapeutic resistance, including resistance to radio- and chemotherapies (95, 96). Furthermore, T-IgG accumulation is enriched in castration-resistant prostate cancer, suggesting possible additional signal involvement in resistance to hormonal therapy (81). In a drug-resistance study of oral carcinoma, silencing T-IgG sensitized tumor cells to cisplatin and significantly reduced cell viability (95). Although this mechanism has not yet been confirmed *in vivo*, it has been hypothesized that activation of Src by PTP-BAS may facilitate drug resistance. This hypothesis is consistent with previous data describing a potential relationship between pharmacological PI3K/AKT inhibition and increased cisplatin sensitivity (97).

In addition to chemotherapy, radiation therapy is frequently used and functions through the application of ionizing radiation to induce DNA double-stranded breaks (DSBs) (98). It is proposed that T-IgG acts to reduce DSB via upregulation of DNA repair mechanisms in lung cancer, as visualized by inverse correlation to γ-H2AX markers (96). Additionally, elevation of DNA-PKcs has been tied to T-IgG-mediated upregulation of PI3K/AKT expression, which could potentiate the non-homologous end joining repair pathway (96). However, it must be noted that since the connection between T-IgG and AKT phosphorylation is not fully verified, further research must be conducted to determine whether, as suggested by previous data, integrin-FAK binding or alternative sequences are utilized (12). Holistically, these findings suggest that T-IgG may be a factor in tumor resilience under therapeutic stress, generating the possibility of T-IgG serving as a biomarker in tumor progression (99).

## 5 The aging to cancer continuum

As previously mentioned, aging is marred by systemic instabilities, with age-associated IgG proposed as a biomolecule involved in this decline (34). Specifically, the loss of terminal galactosylation and sialylation on the IgG Fc domain is a likely culprit for the increased binding affinity to activating Fcγ receptors on resident immune cells, effectively diminishing the threshold for chronic inflammatory tone (100).

This chronic, low-grade inflammation is itself an established hallmark of oncogenesis (101). In this environment, the continual signaling of key cytokines such as IL-6, TNF-α, and IL-1β induces an initial burst of oxidative stress, which subsequently triggers the nuclear translocation of NF-κB and phosphorylation of STAT3 (102, 103). This transcriptional activity potentiates the production of a massive secondary storm of reactive oxygen (ROS) and nitrogen species (RNS) through the induction of cyclooxygenase-2 and iNOS, which damage surrounding cellular stability, foster genomic instability, and ultimately promote mutagenesis (104).

As previously shown, the engagement of macrophage Fcγ receptors by age-associated IgG may elicit the expression of IL-6 and TNF-α (9). Furthermore, recent breakthroughs demonstrate that accumulated IgG non-canonically engages macrophage TLR4 to potentiate IL-1β secretion via the NLRP3 inflammasome (105). In the context of tumorigenesis, both are canonical intermediaries which are associated with the recruitment of myeloid-derived suppressor cells (MDSCs), generating a plausible mechanism by which inflammageing may enable tumor-permissive conditions (106). The immunosuppressive nature of the MDSCs regulates the effector function of T cell populations via local release of Arginase-1 and iNOS, effectively hindering T-cell expansion, which are critical to the detection and clearance of malignant cells (107, 108). Similarly, the binding interaction between age-associated IgG and adipose tissue resident macrophages may drive TGF-β mediated fibrosis, further destabilizing the microenvironment (9).

Upon suppression of immune surveillance, pre-malignant cells are then able to proliferate freely. Once the tumorigenic niche is established, T-IgG becomes increasingly detectable within the tumor microenvironment. This intratumoral IgG is most plausibly supplied by infiltrating B cells and plasma cells, although a contribution from malignant cells themselves has also been proposed and remains unresolved (109). The resulting T-IgG may facilitate immune evasion by reducing effector T cell proliferation and restricting CD8+ T cell infiltration via binding inhibitory Siglecs present on local T cells (14). Furthermore, T-IgG has been reported to engage the TLR4-inflammation axis in cervical cancer cell models, an interaction associated with sustained production of IL-6, TNF-α, and IL-1β and a state of chronic oxidative stress and inflammation (77). These findings are corroborated by Wang et al., which associates T-IgG with chronic low-level ROS generation (86). Ultimately the continuous production of T-IgG fortifies pre-existing tumor-permissive conditions, creating a potential positive feedback loop stemming from the accumulation of age-associated IgG.

**Figure 3:**
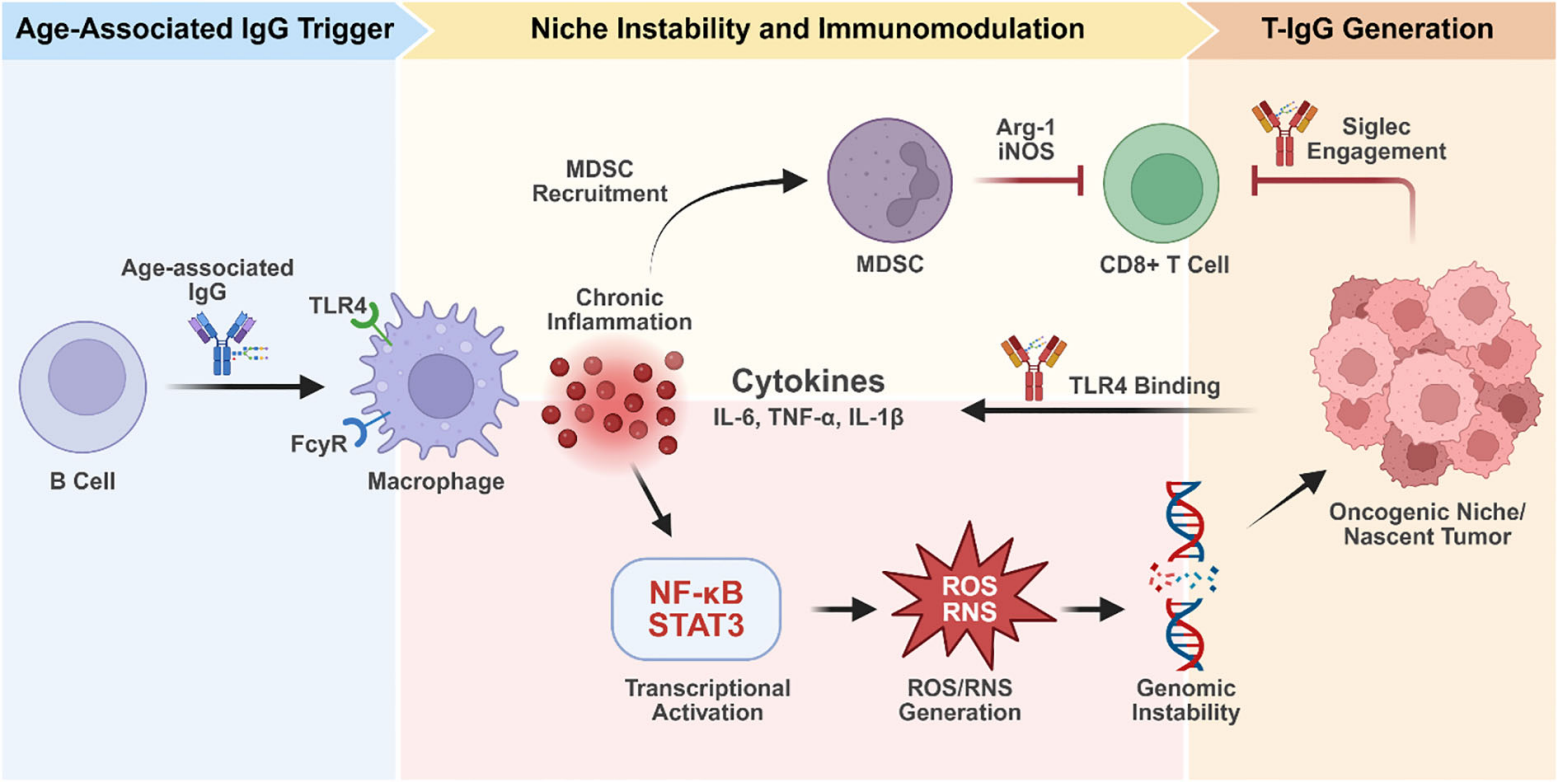
Priming of oncogenic niche by age-associated IgG and T-IgG mediated Inflammatory Amplification. Depicted is the accumulation of age-associated IgG, which establishes an inflammatory environment by engaging macrophage receptors (9, 105). The specific pro-inflammatory cytokines released (IL-6, TNF-α, and IL-1β) trigger NF-κB and STAT3 mediated transcription driving the generation of ROS and RNS which incite a tumor permissive environment (104). Additionally, the aforementioned cytokines drive the recruitment of MDSCs (107). Once the tumorigenic niche is established, T-IgG accumulates within the tumor microenvironment which amplifies immune evasion via inhibitory Siglecs (14). Furthermore, T-IgG hijacks the TLR4-inflammation pathway to sustain the tumor permissive environment (77).

The combined loss of immune surveillance and the sustained inflammatory environment create a niche conducive to the proliferation of malignant cells (102), within which T-IgG accumulates, associating with poor prognosis (110). These contributing factors together may promote pathological tissue remodeling by compromising homeostatic scaffolding, which then primes the emergence of malignancies (**Figure 3**).

## 6 IgG glycosylation as biomarkers

Having identified the aberrant IgG axis as a potential mechanism involved in both inflammaging and tumorigenesis, the clinical imperative shifts toward exploiting these molecular signatures. The distinct glycoforms present on age-associated and T-IgG represent a possibly actionable diagnostic target (20, 111). Here, we evaluate the translational utility of IgG profiling, tracing its application from systemic biological clocks in aging to its potential as a prognostic indicator in oncology (20, 21).

Given the inevitability of aging, there have been longstanding efforts to estimate an individual’s stage of aging by comparing with chronological age. The Lauc laboratory and their collaborators have published extensive literature on this topic, examining glycosylation changes in IgG during aging, providing substantial evidence that these alterations may be used to analyze and predict biological age (20). As previously discussed, certain glycan modifications exhibit consistent trends with age, including decreased galactosylation and sialylation. Following the discovery of these correlations, researchers have attempted to develop novel age-prediction models based on IgG glycosylation patterns across different populations. One such approach, conducted in a Han-Chinese population, resulted in the development of an “aging clock” that accounted for approximately 23% to 45% of variance in chronological age using IgG glycosylation alone (112). Interestingly, a stronger association was observed among the female cohort, which may be linked to hormonal regulation of glycosylation by estrogen (113, 114). A similar approach was conducted using plasma samples from approximately 5,000 individuals across several European countries. The resulting index, termed “GlycanAge” predicted an even greater proportion of variation, up to 58% of chronological age and was also associated with physiological markers of aging (20). However, it is important to contextualize these studies within their cross-sectional frame, as the study cohorts only represent a single temporal window, which may preclude claims of causality until examined longitudinally.

Furthermore, the sensitivity of IgG glycosylation to hormonal influence raises the possibility that Fc glycan profiling could serve as a potential biomarker reflecting hormonal status in addition to immune aging (115). Rather than relying solely on the current strategies that assess endocrine status through direct measurement of circulating hormone concentrations, analysis of IgG glycan patterns may provide insight into early endocrine transition, which may otherwise be overlooked by standard measurement (116). However, further investigation should first clarify key signaling pathways that link hormone levels to IgG glycosylation. Given the occurrence of several malignancies in hormonally distinct environments, additional studies examining the interplay between endocrine signaling and Fc glycosylation may provide valuable insight for both the immune aging and tumor development (115). In future studies, glycosylation-based aging indices should continue to be evaluated for accuracy against traditional ageing markers as well as newer modeling approaches. If validated, IgG glycosylation could emerge as a promising and minimally invasive biomarker that leverages a ubiquitous immune molecule for longitudinal health assessment.

Beyond its involvement in tumor progression, the aberrant expression of T-IgG may serve as a distinct prognostic indicator in clinical oncology (21). Epithelial cancer tissue sample analyses demonstrate that elevated T-IgG levels consistently correlate with advanced tumor staging, heightened metastatic propensity, and prognosis across a spectrum of epithelial malignancies, including breast, gastric, and lung (12, 82, 84, 117). The monoclonal antibody RP215, identified by Lee et al., binds to the CA215 epitope on Asn 162 and offers a novel approach for detecting tumor growth through the accumulation of T-IgG in the TME, which is hypothesized to increase alongside tumor growth (21, 111). However, it should be noted that much of the evidence supporting the conclusion is derived from retrospective analysis of histological tissue samples collected postmortem. Such studies may be susceptible to hindsight bias, inconsistent sampling, and limited representation of broader patient populations (118). Despite early promises demonstrated in published studies, additional validation across diverse cancer morphologies is required before T-IgG can be implemented in clinical practice.

## 7 IgG-based therapeutics in aging and cancer

Given the potential impact of aberrant IgG on both tissue senescence and tumor resilience, disrupting this pathogenic axis represents a compelling therapeutic frontier. However, therapeutic approach is context dependent, whether focusing on systemic glycome modification for age-associated IgG, or the targeted removal of cancer-promoting T-IgG.

### 7.1 Systemic glycome recalibration via metabolic and enzymatic interventions

**Figure 4:**
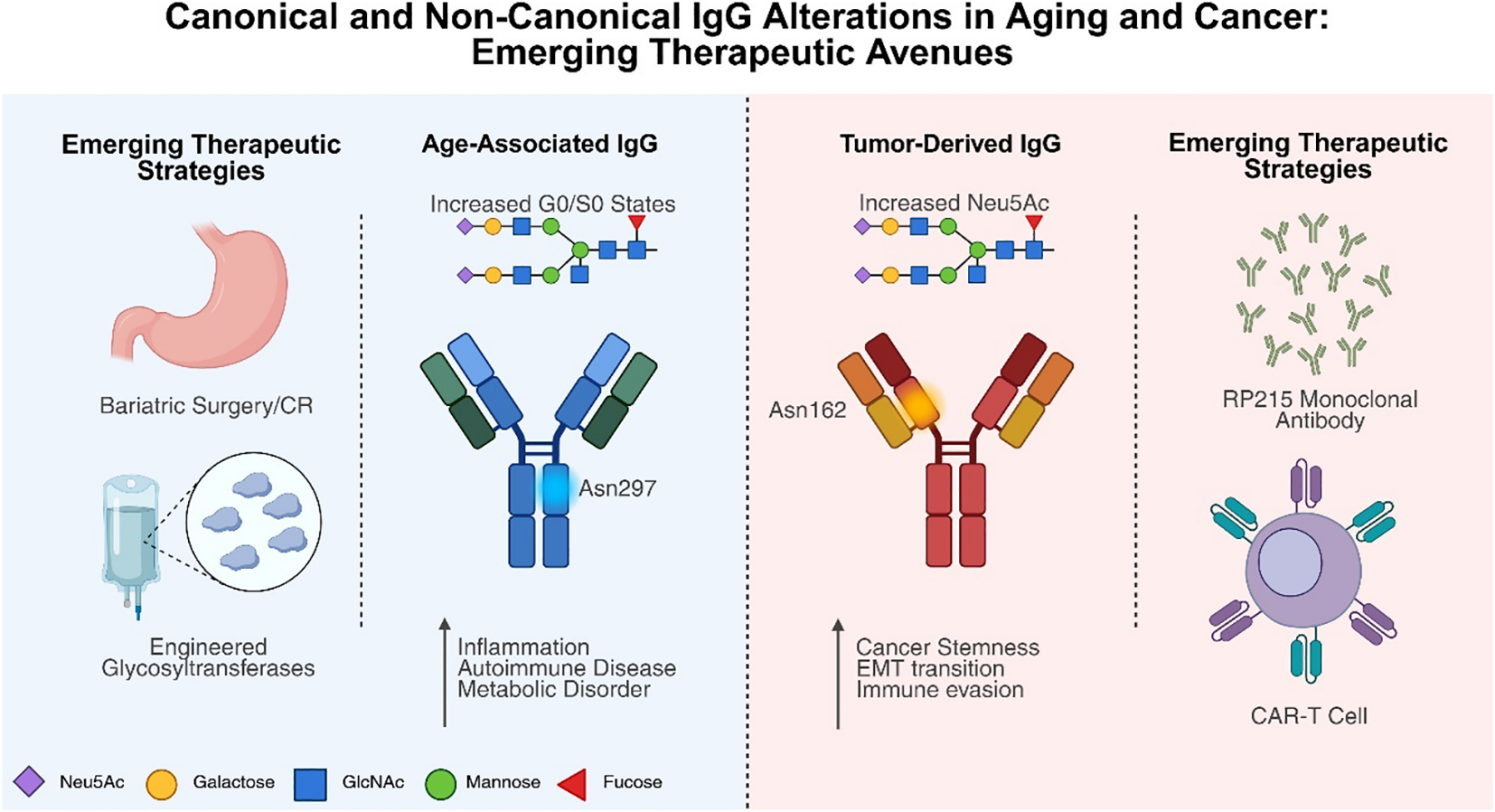
Canonical and non-canonical IgG N-glycosylation in aging and cancer: emerging therapeutic strategies. Differing glycosylation patterns in aging and cancer are depicted. Left: Age-associated IgG glycosylation phenotype in association with systemic inflammation and autoimmune diseases (38, 119). Right: Non-canonical Asn162 glycosylation associated with T-IgG contributes to cancer progression (75). Emerging therapeutic strategies targeting age-associated IgG glycosylation include adipose tissue reduction and engineered glycosyltransferases (22, 120, 121). Targeted interventions against T-IgG include the RP215 monoclonal antibody and CAR-T cells directed against T-IgG epitopes (122, 123).

A major characteristic of aged IgG is its altered Fc glycosylation profile. Given the functional consequences of these changes, an important question is whether age-associated IgG glycosylation is reversible. Calorie restriction (CR), which has been extensively studied, and is thought to potentially slow aspects of aging, has been shown to influence the IgG N-glycome (**Figure 4**) (22, 124). Current evidence suggests that reduced caloric intake may decrease agalactosylated glycans while simultaneously increasing terminal galactosylation, potentially alleviating some of the inflammatory burden associated with chronic inflammation (124). However, findings regarding the significance of these glycosylation changes are not uniform across studies, with some conflicting results reported, indicating the need for further investigation (125).

Beyond caloric intake, direct manipulation of body fat through bariatric surgery has also been shown to affect IgG glycosylation in a similar manner, reducing G0 glycans while increasing IgG sialylation (120). The findings suggest that the glycosylation state of IgG may be linked to body mass index, a relationship that has been previously hypothesized (126). Furthermore, hormonal balance, particularly estrogen levels are known to influence IgG glycosylation (115). This observation raises the possibility that researchers may be able to leverage exogenous hormone administration to influence IgG glycosylation and potentially rejuvenate aging-associated biomarkers. The addition of testosterone has been investigated in relation to glycan age, with findings suggesting reduction in G0 glycans and increased sialylation (127). However, because this study was conducted in obese and hypogonadal patients, the baseline physiological state of the patients was not fully representative of the general population, highlighting a common limitation in studies involving exogenous hormone administration.

In contrast to treatments that rely on the modifying existent tissue, researchers have also sought to address the issue of age-related changes in IgG by directly targeting glycosylation (128). Through the administration of exogenous glycosyltransferases, it may be possible to artificially increase sialylation within the N-glycome. Using this approach, investigators have reported attenuation of pathogenic IgG activity in autoimmune diseases (**Figure 4**) (121). The proposed mechanisms are thought to parallel aspects of intravenous immunoglobulin (IVIG) therapy, potentially involving DC-SIGN and STAT6 signaling pathways that promote anti-inflammatory FcγRIIb expression (121).

Although this mechanism is not universally accepted and remains incompletely resolved, advances in glycoengineering have demonstrated that modifying IgG glycosylation can alter antibody effector function. For example, afucosylation can enhance ADCC, whereas increased sialylation can promote anti-inflammatory signaling pathways (129). These findings support the potential for therapeutically administered IgG with selectively engineered Fc glycosylation to modulate the effects of endogenous age-associated IgG through preferential FcγR interactions.

However, practical implementation of these approaches remains distant. Significant challenges persist, including the use of externally sourced IgG and uncertainties regarding its stability and efficacy after administration (129). While these strategies aim to revert age-associated IgG modifications, similar efforts to sequester T-IgG in cancer were being conducted (78).

### 7.2 Depletion of T-IgG as a therapeutic

Cancer represents a major clinical challenge given its high mortality rates and prevalence, driving the continuous search for effective therapeutics (101). Following the discovery of T-IgG as a molecule associated with cancer proliferation and survival, several studies have investigated whether its removal could yield therapeutic benefits (122, 130). However, targeting the IgG glycome in cancer may be significantly challenging due to the incomplete field of research mapping the exact differences and their interactions in immunology (19, 94, 131). This limitation likely reflects the ability of the tumor to reprogram glycosylation-related enzymes and the strong context dependency of glycosylation patterning in cancer (132).

To date, one of the most promising strategies for targeting T-IgG involves the monoclonal antibody RP215, which binds selectively to T-IgG (78). This antibody demonstrates preferential recognition of T-IgG in tumor samples and has shown limited cross-reactivity with normal IgG, suggesting potential therapeutic application for selectively capturing or targeting T-IgG, although its clinical impact remains uncertain (**Figure 4**) (123).

Similarly, with the emergence of chimeric antigen receptor (CAR) T-cell therapy, efforts have been made to design CAR T-cell targeting sialylated T-IgG (**Figure 4**). Ding et al. developed a prototype CAR T-cell that demonstrated therapeutic potential to HER2-targeted CAR T cells while exhibiting reduced off-target cytotoxicity in bladder cancer cells (122). However, even under the assumption that RP215 and CAR T-cell therapies are minimally harmful to healthy cells, the administration of either approach alone has not yet been clinically evaluated and requires further study. Nevertheless, the use of T-IgG-depleting agents as an adjuvant to established treatment regimens represents a potential strategy for tumor sensitization and may open new therapeutic avenues (122).

Another example involves the use of protoporphyrin IX (PPIX), a naturally occurring metabolite in the heme synthesis pathway that can be artificially upregulated in tumors through administration of its precursor, 5-aminolevulinic acid. When active at an appropriate wavelength, PPIX fluoresces and can serve as a visual aid during surgical procedures (133). T-IgG is relevant in this context, as its depletion has been shown to increase PPIX accumulation in colorectal cancer models. Consequently, RP215 may offer a potential strategy to enhance PPIX accumulation by binding and sequestering T-IgG, thereby increasing the intensity of fluorescence-based surgical imaging (130).

Collectively, the potential therapeutic applications targeting aberrant IgG remain largely exploratory and are far from exhaustive. To date, relatively few clinical trials have investigated IgG-directed interventions in aging populations, while most studies in cancer remain limited to *in-vitro* models (122, 123). Nevertheless, research involving RP215 represents a promising avenue that could complement existing cancer therapies. Similarly, strategies aimed at addressing age-associated IgG alterations, such as calorie restriction have been associated with a shift in the IgG glycome toward a more youthful profile, potentially mitigating aspects of inflammaging and disease susceptibility (22, 78, 124).

## 8 Conclusions and future perspectives

Despite the differences in aging and cancer physiology, there is a notable overlap in immunological features, particularly involving dysregulated effector function and the reduced immune clearance. Across both aging and cancer, IgG remains a prominent component that undergoes substantial post-translational modification to its glycan structure. The resulting variants have been suggested to influence surrounding tissue physiology, as observed in age-related inflammation and in tumor progression (19, 125).

In the context of aging, deterioration of B cells has been shown to produce IgG variants that differ from their traditional counterparts, potentially impairing the tissue microenvironment (5, 9, 38). A growing body of evidence further supports the role of aged IgG in modifying FcγR signaling in immune cells as a potential mechanism underlying these effects (18, 60). Viewed through this lens, IgG exhibits several similarities with cancer biology, although the severity and clinical implications differ substantially between the two conditions.

Whether age-associated IgG predicts or drives spontaneous cancer remains unclear, but comparing IgG in aging and cancer is instructive. T-IgG reportedly shows atypical Asn 162 glycosylation and, in some studies, a restricted repertoire; its source is debated in the field (74), though it is most plausibly supplied by the tumor-infiltrating B-cell and plasma-cell compartment. These characteristics have been proposed to contribute to cancer survival and progression (13, 16). The signaling pathways implicated in these processes frequently converge on key oncogenic networks, including FAK/Src, PI3K/AKT, and MEK/ERK signaling (12, 80, 85).

Additionally, recent findings describing interactions between T-IgG and molecules such as c-Met and integrin α6β4 suggest that T-IgG may support tumor cell plasticity and adaptability (13, 83). These nontraditional interactions underscore the complexity of IgG-mediated processes in both aging and pathologies. Altered IgG glycosylation has been associated with chronic inflammation and metabolic dysregulation, while T-IgG in cancer has been linked to treatment resistance, including chemotherapy and radiotherapy resistance (9, 95, 96).

Although multiple studies have reported T-IgG as a tumor-intrinsic product, the translation of these findings to complex *in-vivo* systems remains limited (75). As such, it is theoretically possible that the atypical IgG originates from the tumor microenvironment B cells rather than the malignant cells themselves. If this were to be the case, the paradigm would shift from an intrinsic oncogenic-signaling-axis to an extrinsic based crosstalk. Importantly, this does not interfere with the underlying principle of our proposed aging to cancer continuum, rather, in such a scenario, the case maintains the same core underlying principle. The accumulation of age-associated IgG induced inflammation in addition to an immunosuppressive niche via irregular FcγR engagement may serve as predecessors to the TME specific T-IgG (9, 38). Regardless of whether the pathogenic IgG is a consequence of intrinsic tumor-cell synthesis or supplied by an age-associated infiltrating B cell population, the existing pro-inflammatory niche and blunted immunity remain well established contributors to cancer development and progression (101). Resolving this controversy through spatial transcriptomics and lineage tracing would be a critical next step for the field.

Furthermore, the development of Fc glycosylated glycan clocks offers a potential approach for estimating biological age, while tumor-selective glycan epitopes such as CA215 may provide additional prognostic opportunities in oncology (111, 112). In addition, studies monitoring the efficacy of RP215, a monoclonal antibody to the epitope, were seen to reduce tumorigenesis in tumor cell samples, posing as a potential therapeutic avenue (117). Moving forward, continued investigation into ligand-receptor interactions and downstream signaling pathways will be essential for establishing causal relationships between IgG modification and immune function. Likewise, further exploration of strategies to modulate IgG glycosylation will be critical for determining their safety and therapeutic potential in addressing age-related tissue dysfunction and cancer progression.

In conclusion, both fields remain at relatively early stages of discovery and lack comprehensive mechanistic validation, particularly regarding age-associated IgG (18, 75). Nevertheless, increasing interest in IgG as a functional component of host physiology is drawing greater attention to its potential biological significance. In this context, IgG may serve as a useful bridge between aging and cancer, as it is both measurable and potentially therapeutically actionable (20, 111).
